# Roux-en-Y gastric bypass and parastomal hernia repair: case report of concurrent operation in comorbid patient

**DOI:** 10.1016/j.ijscr.2020.05.024

**Published:** 2020-05-21

**Authors:** Alexander Khitaryan, Ismail Miziev, Arut Mezhunts, Camil Veliev, Raisa Zavgorodnyaya, Alexey Orekhov, Vasily Kislyakov, Anastasiya Golovina

**Affiliations:** a Private Healthcare Institution Clinical Hospital “RGD-Medicine”, Varfolomeeva Street 92, Rostov-on-Don, Russian Federation; b FSBEI HE Rostov State Medical University of the Ministry of Health of the Russian Federation, Nakhichevansky Lane 19, Rostov-on-Don, Russian Federation; c FSBEI HE Kabardino-Balkarian State University named after Berbekov H.M., Chernyshevskiy Street 173, Nalchik, Russian Federation

**Keywords:** Parastomal hernia, Roux-en-Y gastric bypass, Bariatric surgery, IPOM, Case report

## Abstract

**Introduction:**

The treatment of parastomal hernias remains one of the most relevant issues in coloproctology and general surgery due to its high recurrence rate of 5 to 50%. An increase in the number of overweight people has led to the fact that at least 25% of patients with parastomal hernias are obese and have severe concurrent disorders.

**Presentation of case:**

A 69-years old woman with 12 × 15 cm parastomal hernia, grade 3 obesity and type 2 diabetes mellitus underwent concurrent laparoscopic IPOM hernia repair and Roux-en-Y gastric bypass. The patient was discharged on the 5^th^ postoperative day. After 12 months the patient lost 42 kg, BMI = 28.3 kg/m^2^, had a complete remission of diabetes, and no signs of parastomal hernia.

**Discussion:**

Symptomatic parastomal hernias, accompanied by pain, episodes of incarceration, impaired evacuation of intestinal contents through the ostomy, and dermatitis require surgical intervention. The combination of bariatric surgery and simultaneous hernioplasty is a standard intervention approved in the respective guidelines. At the same time, in the case of parastomal hernias after colorectal operations, the risk of encountering a serious adhesion process can complicate laparoscopic surgery. In obese patients with type 2 diabetes mellitus, it is recommended to perform one of the bypass interventions.

**Conclusion:**

Concurrent bariatric surgery and hernia repair allow the patient to lose more than 70% of excess body weight, reduce the risk of hernia recurrence, and significantly reduce comorbidity. This surgical approach is safe in thoroughly selected patients, who might greatly benefit from it.

## Highlights

• Parastomal hernias have very high recurrence rate after surgical treatment, it ranges from 5 to 50%.
• An increasing in the number of overweight people has led to the fact that 25% of patients with parastomal hernias are obese and have severe concurrent disorders.
• A 69-years old woman with 12 × 15 cm parastomal hernia, grade 3 obesity and type 2 diabetes mellitus underwent concurrent laparoscopic IPOM hernia repair and Roux-en-Y gastric bypass.
• The patient had an uneventful, standard recovery and was discharged on the 5th postoperative day.
• After 12 months the patient lost 42 kg, BMI = 28.3 kg/m^2^, had a complete remission of diabetes and no signs of parastomal hernia.

## 1 Introduction

The treatment of parastomal hernias remains one of the most relevant issues of coloproctology and general surgery. The recurrence rate after surgical treatment is quite high and ranges between 5 to 50%. [1], [2], [3]. Parastomal hernia is clinically manifested by pain and discomfort, thereby significantly reduces the quality of life of the patients and might lead to stoma evacuation disorders, parastomal incarcerations up to the development of bowel obstruction [4]. Hernias significantly discommode ostomy hygiene and can lead to the development of dermatitis [5], [6], [7]. Surgical planning for hernia repair usually requires a multidisciplinary approach, since the main relapse risk factors are comorbidity, the presence of diabetes mellitus, obesity, anemia, and smoking. The best results of surgical repair can be achieved with preoperative correction of concomitant diseases, weight loss, quitting smoking, which requires very high, often unattainable, patient compliance. An increase in the number of overweight people over the past 3 decades has led to the fact that at least 25% of patients with parastomal hernias are obese. This fact requires new solutions in the treatment of the disease [8].

The gold standard in the treatment of obese patients with type 2 diabetes is Roux-en-Y gastric bypass surgery (RYGB). This intervention ensures weight loss and type 2 diabetes correction in more than 70% of cases [9]. The standardization of laparoscopic interventions in bariatric surgery along with the development of surgical equipment made it possible and safe to perform simultaneous operations aimed at reducing the patient's weight and hernia repair [10], [11]. This trend was largely facilitated by the advent of IPOM technology. There are more and more reports in literature about the benefits of IPOM in patients with parastomal hernias.

This report demonstrates the surveillance of a patient with a parastomal hernia, grade 3 obesity, and type 2 diabetes mellitus over a one-year period after successful concurrent laparoscopic IPOM parastomal hernia repair and Roux-en-Y gastric bypass surgery. The patient was treated in a non-governmental clinical hospital. The work is reported in line with the SCARE criteria [12].

## 2 Presentation of case

A 69-years old woman came with complaints of abdominal wall deformation in the ostomy area, constant aching in the same place, evacuation difficulties, and progressive increase in body weight. The patient had undergone extralevator abdominoperineal excision with a permanent colostomy for colorectal cancer pT3N1M0 st. 3A, and 8 courses of polychemotherapy. According to the investigations (computed tomography, carcinoembryonic antigen), the patient was in persistent remission of cancer. She had noticed the protrusion in the colostomy area about 6 months ago. Recently, the protrusion started to rapidly increase and became painful. It was also known that she had been suffering from type 2 diabetes mellitus for 10 years and was receiving oral hypoglycemic agents (biguanides and sulfonylureas). The diabetes was poorly controlled: fasting blood glucose was 6.8 mmol/L, glycated hemoglobin 7.1%, C-peptide 800 ng/ml. A progressive increase in body weight over 2 years was also noted. The height of the patient was 164 cm, weight 118 kg, BMI 43.9 kg/m^2^. A physical examination revealed parastomal hernia 12 × 15 cm in size, causing the deformation of the anterior abdominal wall, significantly increasing with the vertical position of the body, reducible and moderately painful on palpation.

**Fig. 1:**
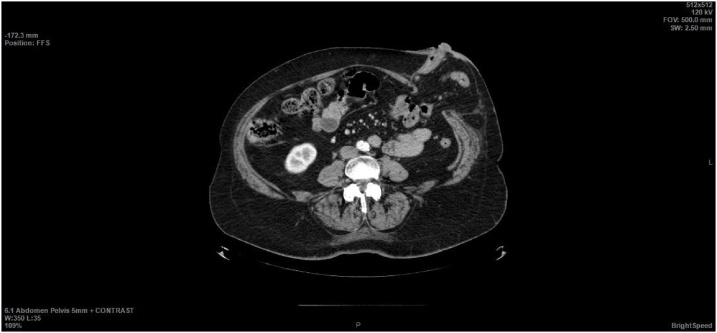
Abdominal CT scan.

The preoperative examination included abdominal ultrasound and CT scan, esophagogastroduodenoscopy and colonoscopy. According to CT (see Fig. 1), there was a hernial protrusion of 96 × 81 mm in size in the colostomy area. The contents of the hernia sac were ileal loops. The dimensions of the hernia gate were 43 × 40 mm. Moderate diffuse liver lesions, detected at abdominal ultrasound, were interpreted as hepatic steatosis.

So, we decided to perform concurrent laparoscopic IPOM hernia repair and Roux-en-Y gastric bypass.

### 2.1 Surgical technique

**Fig. 2:**
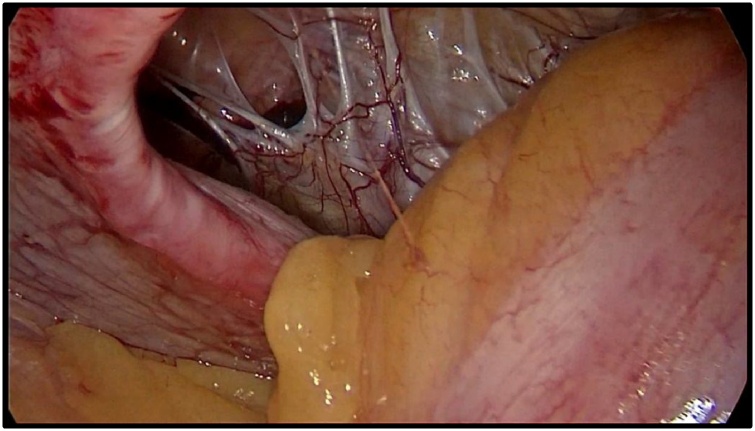
The parastomal hernia, intraabdominal view.

Preoperative preparation included compression stockings and heparin as VTE prophylaxis and antibiotics (2 grams of ceftriaxone and metronidazole). The operation was performed in the supine position with a raised head end and rotation of the operating table on the right side. The first 12 mm trocar was installed in the right hypochondriac region, along the midclavicular line, two 11 mm trocars were installed in the right mesogastric and iliac regions. Abdominal adhesions after the previous laparoscopic and perineal extralevator extirpation of the rectum were insignificant. Then the 30° laparoscope was transferred to the right mesogastric trocar; adhesions between the anterior abdominal wall and bowel around hernia gates were dissected using EnSeal device (Ethicon US, LLC) (Fig. 2).

**Fig. 3:**
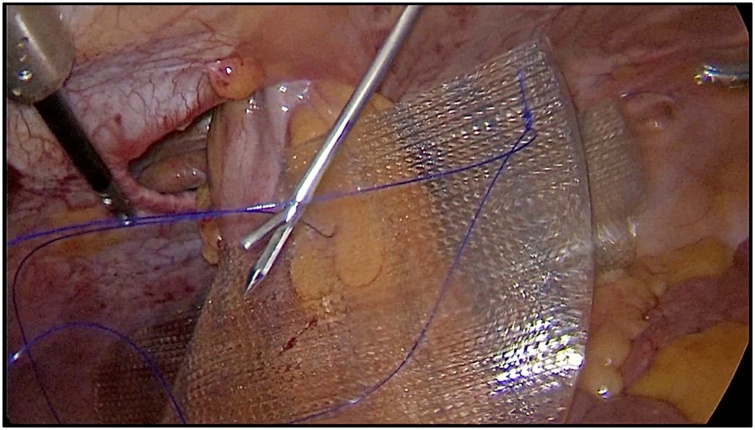
The IPOM mesh placement.

**Fig. 4:**
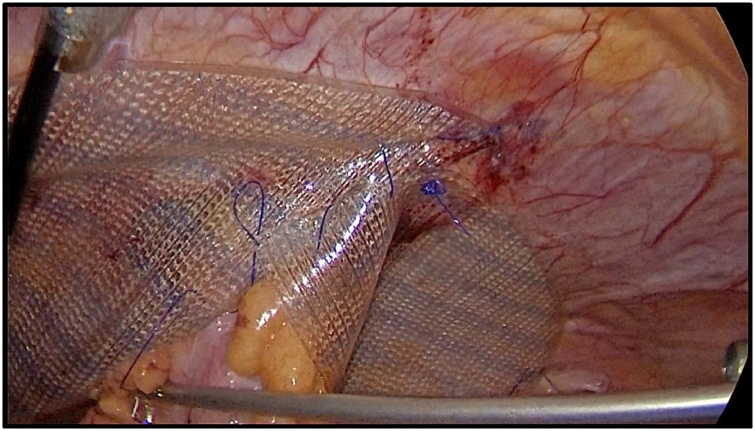
The IPOM mesh fixation.

After hernial sac revision and complete herniolysis, a composite mesh 15 × 20 cm was inserted into the abdominal cavity through a 12 mm trocar. The mesh for IPOM hernioplasty had a 3.5 cm hole in the center for the intestine loop. Using the keyhole technique, the mesh was straightened in the area of the hernial gate with the colon passing through the mesh window and then fixed by 4 transabdominal Prolene 2.0 ligatures sewn to the mesh on each side (Fig. 3). Additional fixation to the peritoneum was carried out by continuous Prolene 2.0 suture along the perimeter of the mesh. Another Prolene 2.0 suture in the area of the window fixed the mesh along with colon and mesentery for the prevention of mesh displacement and internal infringement (Fig. 4).

Before the second stage of the operation, we ensured that the small intestine loops were mobile. Then 4 additional trocars were installed: 11 mm supraumbilical, 12 mm in the left hypochondrium along the midclavicular line, 11 mm along the left anterior axillary line and 5 mm in the epigastric region. The patient was transferred into the Fowler's position. After that the greater omentum was divided using EnSeal device and the stomach was pulled caudally. Then a gastric section was performed 4 cm below gastroesophageal junction using a 45 mm yellow load of Echelon linear stapler (Ethicon US, LLC). Following the mobilization of the superior and posterior walls of the gastric fundus, a 36-Fr gastric lavage tube was introduced by the anesthesiologist. The gastric section was then completed with additional firings of 60 mm and 45 mm yellow loads of Echelon. Next the jejunum 70 cm distal to the ligament of Treitz was pulled to the stomach and fixed with continuous hand suture. A gastrotomy and enterotomy were performed using electrocautery to create orifices of up to 35 mm. Then handsewn anastomosis with continuous single-row V-loc suture was applied. Then an alimentary limb 140 cm long was formed and a handsewn anastomosis with a single-row continuous suture PDS 2.0 was applied. The impermeability of the anastomoses was checked by pneumohydraulic test. Next the afferent loop was cut at 2 cm from anastomosis with a 45 mm yellow load of linear stapler. The operation was finished by thorough hemostasis and abdominal draining. The silicone drains were left for 12 hours.

The surgery was performed by the team, consisted of 3 experienced in laparoscopy bariatric surgeons. On the 5th postoperative day, the patient was uneventfully discharged in satisfactory condition.

**Fig. 5:**
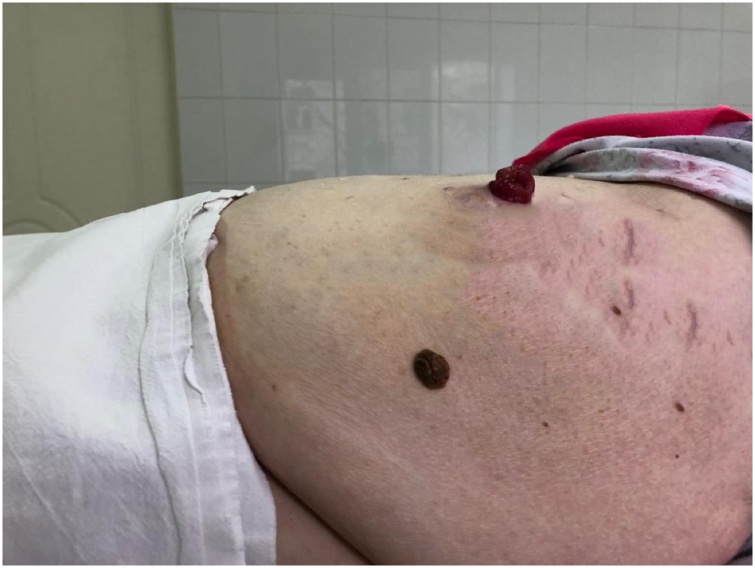
The appearance of the abdominal wall 12 months after the surgery.

*After 12 months.* The patient lost 42 kg, BMI = 28.3 kg/m^2^. Complete remission of type 2 diabetes mellitus was observed: the fasting blood glucose level was 5.5 mmol/L; glycated hemoglobin 5.8%. No signs of parastomal hernia were detected (Fig. 5).

## 3 Discussion

Patients suffering from parastomal hernias are at particular risk for the development of infectious complications and relapses of the disease. It is quite difficult to achieve adequate weight loss before surgery with conservative measures, especially in patients with a BMI of more than 35 kg/m^2^. Symptomatic parastomal hernias, accompanied by pain, episodes of incarceration, impaired evacuation of intestinal contents through the ostomy, parastomal dermatitis require indispensable surgical intervention [12].

The combination of bariatric surgery and concurrent hernioplasty is a standard intervention approved in the respective guidelines [6]. At the same time, in the case of parastomal hernias after colorectal operations, the risk of encountering a serious adhesion process and damage to the small intestine can complicate laparoscopic surgery. In obese patients with type 2 diabetes mellitus, it is recommended to perform one of the bypass interventions. At the same time, in the reported case the conditions for this intervention were favorable due to the insignificant abdominal adhesions and the possibility of free traction of intestinal loops. Otherwise, it would be better to perform a restrictive intervention, for example, sleeve gastrectomy.

The presented surgical technique of Roux-en-Y gastric bypass is standardized in our clinic and does not increase the risk of bariatric procedures. We widely use IPOM hernia repair but consider appropriate to use it for defect sizes up to 40 cm^2^.

## 4 Conclusion

The surgical tactics in patients with parastomal hernias should be strictly individual, taking into account the opinion of the multidisciplinary team. Recommendations for nutrition, ostomy care, compensation for type 2 diabetes mellitus, preoperative weight loss can play a significant role in the improvement of the condition of the patient. However, concurrent bariatric surgery and hernia repair allow the patient to lose more than 70% of excess body weight and decrease the risk of hernia recurrence, significantly reducing comorbidity.

## Conflict of interest

The authors declare no conflicts of interest. The authors have no financial, consultative, institutional and other relationships that might lead to bias or conflict of interest.

## Funding

This research did not receive any specific grant from funding agencies in the public, commercial, or not-for-profit sectors.

## Ethical approval

Ethical approval has been exempted by our institution.

## Informed consent

Written informed consent was obtained from the patient for publication of this case report and accompanying images. A copy of the written consent is available for review by the Editor-in-Chief of this journal on request.

## Author contribution

Khitaryan А. – conceptualization, funding acquisition, investigation, methodology, project administration, resources, supervision, verification, writing original draft, writing review & editing.

Miziev I. – conceptualization, funding acquisition, investigation, methodology, project administration, resources, supervision, verification, writing original draft, writing review & editing.

Mezhunts A. – data curation, formal analysis, software, visualization, writing original draft, writing review & editing.

Veliev K. – data curation, investigation, writing original draft, writing review & editing.

Zavgorodnyaya R. – data curation, investigation, writing original draft, writing review & editing.

Orekhov А. – conceptualization, writing original draft, writing review & editing.

Kislyakov V. – data curation, formal analysis, software, visualization, writing original draft, writing review & editing.

Golovina A. – conceptualization, formal analysis, project administration, resources, software, visualization, writing original draft, writing review & editing.

## Registration of Research Studies

None. This publication is neither ‘first-in-man study’ nor a research.

## Guarantor

Khitaryan А.G.

Golovina A.A.

## Provenance and peer review

Not commissioned, externally peer-reviewed.
